# The Cure: Design and Evaluation of a Crowdsourcing Game for Gene Selection for Breast Cancer Survival Prediction

**DOI:** 10.2196/games.3350

**Published:** 2014-07-29

**Authors:** Benjamin M Good, Salvatore Loguercio, Obi L Griffith, Max Nanis, Chunlei Wu, Andrew I Su

**Affiliations:** ^1^The Scripps Research InstituteDepartment of Molecular and Experimental MedicineLa Jolla, CAUnited States; ^2^Washington University School of MedicineDepartment of MedicineSt Louis, MOUnited States

**Keywords:** breast neoplasms, gene expression, artificial intelligence, survival analysis, crowdsourcing, Web applications, computer games, collaborative and social computing systems and tools, supervised learning, feature selection

## Abstract

**Background:**

Molecular signatures for predicting breast cancer prognosis could greatly improve care through personalization of treatment. Computational analyses of genome-wide expression datasets have identified such signatures, but these signatures leave much to be desired in terms of accuracy, reproducibility, and biological interpretability. Methods that take advantage of structured prior knowledge (eg, protein interaction networks) show promise in helping to define better signatures, but most knowledge remains unstructured. Crowdsourcing via scientific discovery games is an emerging methodology that has the potential to tap into human intelligence at scales and in modes unheard of before.

**Objective:**

The main objective of this study was to test the hypothesis that knowledge linking expression patterns of specific genes to breast cancer outcomes could be captured from players of an open, Web-based game. We envisioned capturing knowledge both from the player’s prior experience and from their ability to interpret text related to candidate genes presented to them in the context of the game.

**Methods:**

We developed and evaluated an online game called The Cure that captured information from players regarding genes for use as predictors of breast cancer survival. Information gathered from game play was aggregated using a voting approach, and used to create rankings of genes. The top genes from these rankings were evaluated using annotation enrichment analysis, comparison to prior predictor gene sets, and by using them to train and test machine learning systems for predicting 10 year survival.

**Results:**

Between its launch in September 2012 and September 2013, The Cure attracted more than 1000 registered players, who collectively played nearly 10,000 games. Gene sets assembled through aggregation of the collected data showed significant enrichment for genes known to be related to key concepts such as cancer, disease progression, and recurrence. In terms of the predictive accuracy of models trained using this information, these gene sets provided comparable performance to gene sets generated using other methods, including those used in commercial tests. The Cure is available on the Internet.

**Conclusions:**

The principal contribution of this work is to show that crowdsourcing games can be developed as a means to address problems involving domain knowledge. While most prior work on scientific discovery games and crowdsourcing in general takes as a premise that contributors have little or no expertise, here we demonstrated a crowdsourcing system that succeeded in capturing expert knowledge.

##  Introduction

### Molecular Predictors for Breast Cancer

Breast cancer is the most common form of cancer in women [1]. It has been studied extensively with genomic technologies, with many attempts to devise molecular predictors of clinical outcomes [2-4] and drug response [5]. If successful, tests derived from these predictors would pave the way toward personalized therapy and better care. While much progress has been made, including several commercially available tests [6], molecular predictors consistently show lower than desirable accuracy, degrade in performance in subsequent validation studies, identify different gene sets in every permutation, and often have no discernable biological rationale [7].

Here, we address the challenge of predicting survival based on gene expression and copy number variation. Given a database of these genomic measurements and associated clinical outcomes, the objective is to produce a classifier that will accurately separate the patients into two classes, those that survive beyond ten years from initial diagnosis, and those that do not. Any such attempt at class prediction based on high-throughput (eg, microarray) data is technically challenging because of the very large number of potential features [8]. Typical datasets provide measurements for tens of thousands of genes, and each gene is a potential predictive feature for use in a classifier. The individual members of optimal feature sets work synergistically, displaying relationships that make the group more useful for prediction as a whole than any individual unit. The space of possible feature combinations is too large to explore exhaustively and, even if it were, the tests available for evaluating feature set quality are not precise. As a result, researchers rely on heuristics and, increasingly, on prior knowledge to identify good feature groups.

Recent gene selection methods are driven by structured prior knowledge in forms such as protein-protein interaction networks [9,10], pathway databases [11,12], and information gathered from pan-cancer datasets [13]. These methods guide the search for predictive gene sets toward cohesive groups related to each other, and to the predicted phenotype through biological mechanism. In doing so, they have improved the stability of the gene selection process and the biological relevance of the identified signatures. These techniques hint at the potential of strategies that marry a top-down approach based on established knowledge with a bottom-up approach based directly on experimental data, but they have not yet produced substantially greater accuracy than other approaches. This may be due in part to a scarcity of relevant structured knowledge with which to compute.

Since the year 2000, more than 166,000 publications related to breast cancer have been added to PubMed [14]. Within that body of literature, and in the minds of those that have created and consumed it, lays a wealth of knowledge relevant to selecting gene sets for survival prediction. Here, we explore a crowdsourcing approach for tapping into that knowledge.

### Crowdsourcing

Crowdsourcing processes take tasks traditionally performed by individuals or small groups and reformulate them such that large numbers of people can participate in their completion. There are many instantiations of the crowdsourcing paradigm [15], here we focus on just one, games with a purpose (GWAP) [16]. GWAPs incentivize large scale work by translating the required labor into elements of games. The games are played for fun, for learning, and to aid in achieving the underlying purpose. Popular GWAPs within biology include Foldit for protein folding [17], Phylo for multiple sequence alignment [18], and MalariaSpot for image analysis [19]. Here we introduce a GWAP for genomic feature selection called The Cure.

Our high-level objective is to identify genes that can be used to build improved prognostic predictors for breast cancer. Our hypothesis is that, if aggregated effectively, the collective knowledge, reading, and reasoning ability of a large community could help to identify genes that are useful in constructing robust classifiers, but might be hidden from purely data driven approaches. In striving to achieve that aim, we conducted the study described here to assess the feasibility of the use of an open, online game (The Cure) in capturing pertinent, expert-level biomedical knowledge.

The central questions addressed are: (1) How many people, of what levels of expertise, would play a game oriented around gene selection for breast cancer survival prediction and why? (2) Would it be possible to extract a gene ranking from the results of play that reflected biomedical knowledge? That is, could the game act as a portal for expert-level knowledge transfer? And (3) could the gene ranking captured through the game be used to generate classifiers that perform well in cross-dataset evaluations?

The null hypotheses are that: (1) no one would play, (2) the results of their play would not yield discernible biological knowledge, and (3) any gene ranking produced would be no better than random. Below we discuss the design of the game, and then present results from one year of open play that shed light on each of the questions posed above.

## Methods

### Game Design

The Cure is a Web application consisting of the pages home, login, board selection, game, and help. The home page provides information about the project and the game, and allows users to either log in or create accounts. Users must create an account to play. During account creation, users must select a username and password, and have the option of entering an email address and answering three short survey questions: (1) “Most recent academic degree?”, (2) “Do you consider yourself knowledgeable about cancer biology?”, and (3) “Do you consider yourself a biologist?”.

### Training

When players first register, they are presented with a training stage that must be passed before they enter the main game area. The training stage consists of four “boards” containing 2 to 4 features common to animals such as “number of legs, breathes air, produces milk, etc”. To complete the level, the player must select the features that can best be used to discriminate between mammals and other classes of animal, before the games automated opponent “Barney” beats them to it. This task was chosen as a way to introduce the dynamics of the game, and to get across the idea of feature selection for classification on a straightforward problem.

### Game

**Figure 1:**
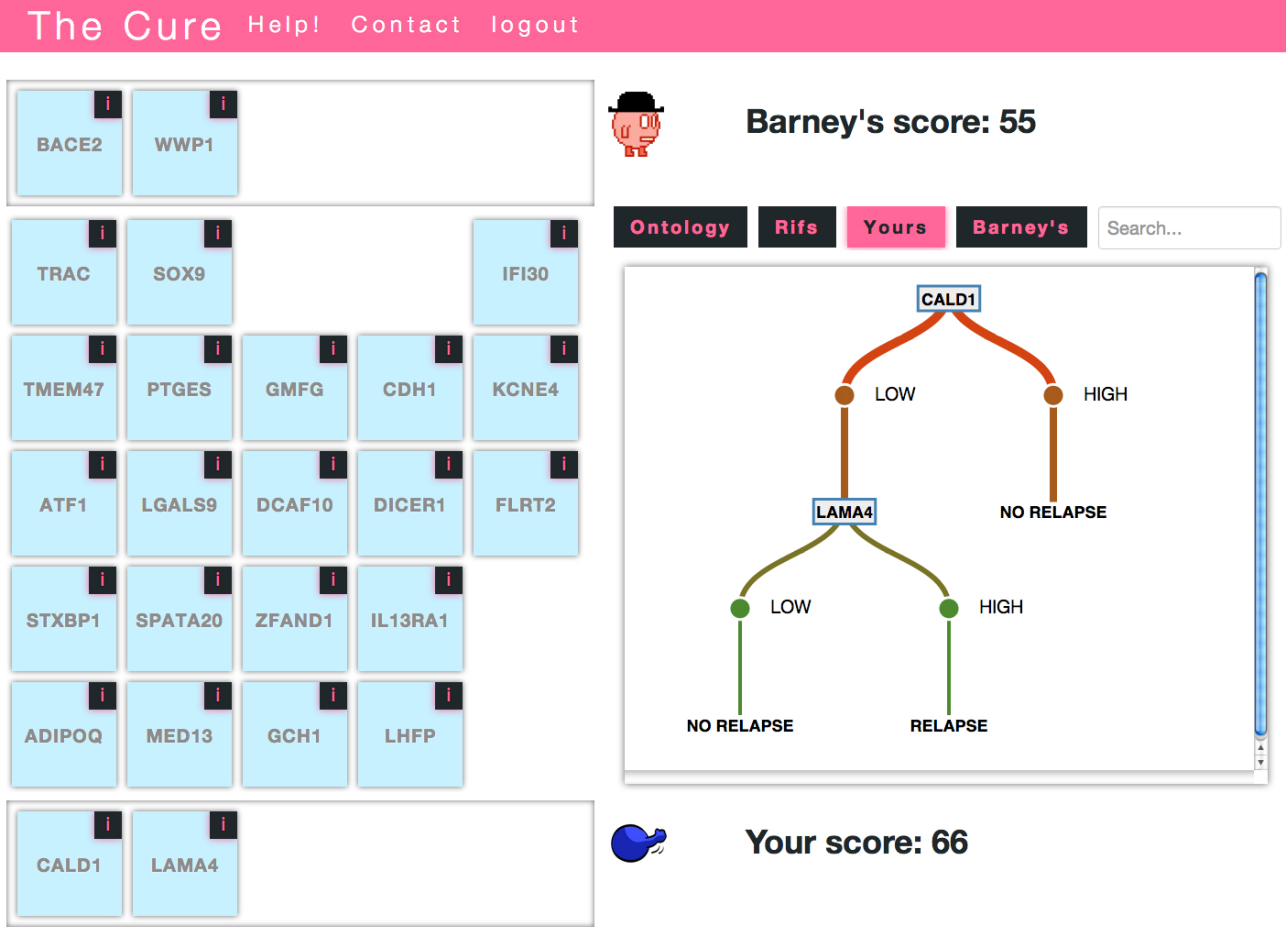
The Cure game. The figure shows a game in progress in which both players have completed 2 of the 5 turns. Players alternate turns, taking a gene card from the board and adding it to their hand. The player with the highest score after 5 turns is the winner. The tabbed display provides gene annotations ("Ontology", "Rifs") and views of decision trees constructed by the system using the selected genes. The scores reflect the predictive power of the selected genes. The system produces these scores by using data associated with the selected genes to train and test a decision tree classifier. The scores are the accuracy of these inferred classifiers.

After training, the player is presented with boards containing 25 different genes (Figure 1 shows an example board). The objective of each game is to choose a set of 5 genes that produces a better decision tree classifier than that of the automated opponent “Barney”. The players, the human player and Barney, alternate turns, taking a gene card from the board and placing it in their hand, with the human player always going first. Once a card is taken from the board, it cannot be put back, and the other player cannot take it. The score for the final 5 card hand determines the winner of the game. Note that each time a board is rendered, the locations of the genes are randomized to prevent bias.

### Gene Annotations

**Figure 2:**
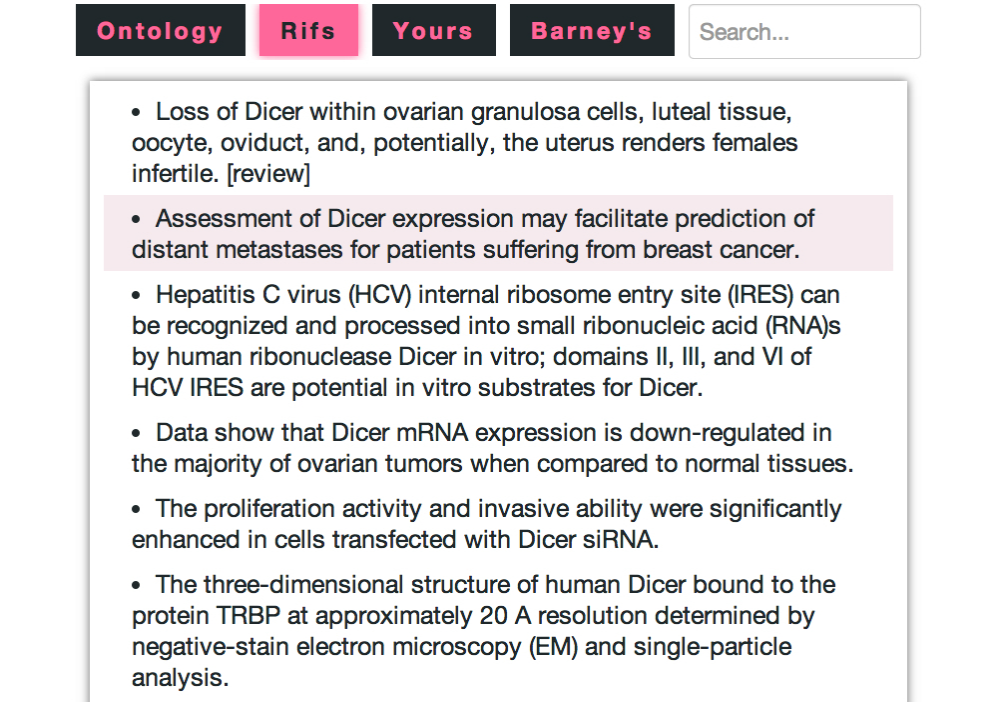
The Gene Rifs tab showing information about the Dicer gene. Gene Rifs provide textual descriptions of gene function extracted from abstracts. These can be used to gain insights into the possible connections between the gene and breast cancer prognosis, and thus can help players to intelligently select genes in the game.

Dragging the mouse over each gene provides the player with information including: (1) a summary description from Unigene, (2) Gene Ontology annotations, and (3) snippets of text related to the gene from The United States National Center for Biotechnology Information’s Gene Rifs (Figure 2 shows these tabs). All of the annotations contain hyperlinks that the players can follow for more information. A search interface allows the player to find genes on the board based on the text in their related annotations. Coupled with the player’s biological knowledge, this information helps the player make informed guesses about which genes from the board might make the most useful predictors.

### Scoring

Each time a card is added to a player’s hand, the game server scores the hand by evaluating the combined predictive performance of the genes it contains. To accomplish this evaluation, the server uses a gene expression dataset containing samples classified with long-term (>10 year) survival status. In each evaluation, the server uses data from just the genes in the player’s hand to train and test a decision tree classifier. The score for the hand is the accuracy returned by a cross-validation experiment. In machine learning parlance, this is known as a “wrapper” feature set evaluation scheme [20]. A simplified decision tree created using all of the available training instances, but just the selected genes, is displayed for the player and their opponent (Figure 1 and Multimedia Appendix 1 for additional details on the implementation of the scoring process). If the player loses, they are not awarded any points; they may play the board again or select a different board to play. If they win, their score is determined based on the accuracy of their winning tree. Within each round, player scores are cumulative. The more games they win, the higher their score. The player’s score is displayed on the board selection page along with its global rank and the current top 10 scores.

### Board Selection

**Figure 3:**
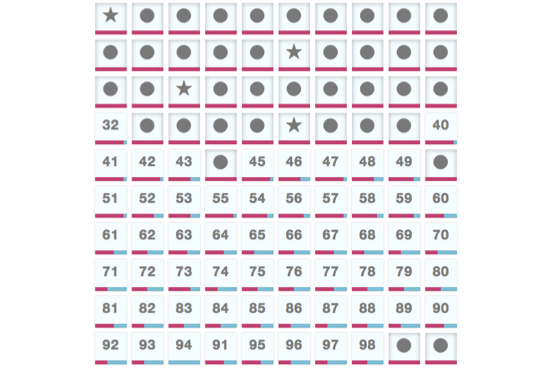
The board selection view. Stars indicate boards the active player has completed, circles indicate boards that have been completed by a sufficient number of different players, and numbers indicate open boards. The pink progress bar indicates how close the community is to finishing the board.

Each round of The Cure consists of a collection of 100 different boards for players to choose from (Figure 3 shows this selection). Each board is composed of a different set of 25 semirandomly selected genes (see Multimedia Appendix 1 for board composition strategies). The boards are arranged in loose order of difficulty, with the easiest boards occupying the lower numbers. The difficulty is assessed based on an estimation of the predictive power of the complete 25 gene set, the more predictive, the easier the board. The goal of the board selection page is to capture both broad and deep coverage of all the boards (and their corresponding gene sets) by the player community. Once a given board has been completed by at least 11 players, it is closed off so that players are forced to select a different board. Any open board can be selected for play. Once a player has completed a particular board, they are not allowed to play it again.

### Purpose

The purpose of The Cure is to translate the knowledge of the players, along with their ability to process textual information, into a ranked list of genes for use in the development of predictors for breast cancer prognosis. This translation is enacted when the players select genes in the game. We record the gene selections, and apply aggregation functions to produce gene rankings that reflect the consensus of the player community.

### Aggregation Function for Gene Ranking

Each time a player selects a gene in a game, they are indicating to the system their intuition of that gene’s relevance for predicting breast cancer survival. That intuition may be based on their knowledge, on inferences drawn from gene annotation information, or solely on random speculation. By aggregating the data collected from many different players across many different games, we tried to eliminate the noise from random clicking and reveal the community consensus with regard to predictive genes.

Given a set of recorded games, our gene ranking method is as follows. For each gene g, we estimate the frequency of selection F(g) as,

F(g)=(S(g))/(O(g))

O(g) equals the number of times the gene g appeared in a played game. Some genes appear on multiple boards, multiple players play all boards, and all occurrences are counted. S(g) is the number of times the gene was selected by the human player.

We then empirically calculated a one-tailed *P* value for each value of F given O through simulations of random game play. The *P* values indicate the chances of observing a value of S or greater given O, assuming that all gene selections were random. Importantly, they allow for comparisons between genes with different numbers of occurrences. For example, the known apoptosis regulator BCL2 gene occurred in 13 played games (O=13), and was selected in 10 of those games (S=10), thus F for BCL2 was 0.77 with *P*<.001. Our simulations stopped at 10,000 iterations per value of O, hence *P* values below .0001 cannot be reported. On the other end of the spectrum, the AARD gene (of unknown function) appeared in 33 played games (O=33), was selected 3 times (S=3), had an F of 0.09 with *P*=.91. Given any collection of played games, we generate gene rankings based on the estimated *P* values for each value of F. We can thus assemble gene sets based on different groups of games as well as different *P* value cutoffs.

### Gene Set Assessments

#### Quality

Given the gene sets produced by this system, we assess quality by: (1) direct comparison to gene sets used in published predictors of breast cancer survival, (2) gene set enrichment analysis, and (3) classifier accuracy.

#### Enrichment Analysis

Enrichment analysis is a widely used statistical technique for assessing the functional roles of gene sets based on their annotations. Given a set of genes with annotations such as Gene Ontology or Disease Ontology associations, these tests estimate the annotation terms that are overrepresented in the gene set. For example, a typical high-throughput experiment may identify a set of 100 or more active genes in a given condition. An enrichment analysis can be used to detect if genes related to a functional category, such as the immune response or a disease group such as cancer, are represented in that set of 100 genes more than they would be expected to by chance. By applying enrichment analysis to the gene sets produced by The Cure player community, we can identify whether genes annotated with terms related to breast cancer or other related diseases or processes are being preferentially selected, as we would expect if the players are not choosing randomly. In principle, it could also unearth interesting new categories of genes selected by the player community.

#### Classifier Accuracy

Finally, we measure the value of the gene sets by using them to construct machine-learning-based classifiers that predict 10 year survival. Given a particular dataset, we eliminate measurements from all genes outside of the set in question, and use the remaining measurements to train and test a predictive model. For the experiments conducted here, we trained support vector machine (SVM) classifiers on gene expression derived datasets, and tested them on independent test sets. We compare the accuracy of the predictors produced using gene sets derived from the game and gene sets used in published survival predictors.

## Results

### Data From One Year of Game Play

The results presented here are derived from games played between September 7, 2012 and September 5, 2013. In that time, 1077 player accounts were created and a total of 15,669 games were played (including training games). There were 9904 games that were played on the cancer datasets. All collected data except personal player information is available, see Multimedia Appendix 2.

### Players and Games Played

Based on the self-reported data collected during registration, the player population was mixed in terms of education, orientation as a biologist, and declared knowledge of cancer. In total, 35.00% (377/1077) of the players had a graduate degree, 28.88% (311/1077) had an undergraduate degree, and 36.12% (389/1077) did not declare any degree. There were 31.94% (344/1077) of the players that considered themselves biologists, while 62.67% (675/1077) did not, with 5.39% (58/1077) not responding. There were 32.96% (355/1077) of the players that declared that they were knowledgeable about cancer biology, 60.35% (650/1077) did not, and 6.69% (72/1077) declined to respond.

**Figure 4:**
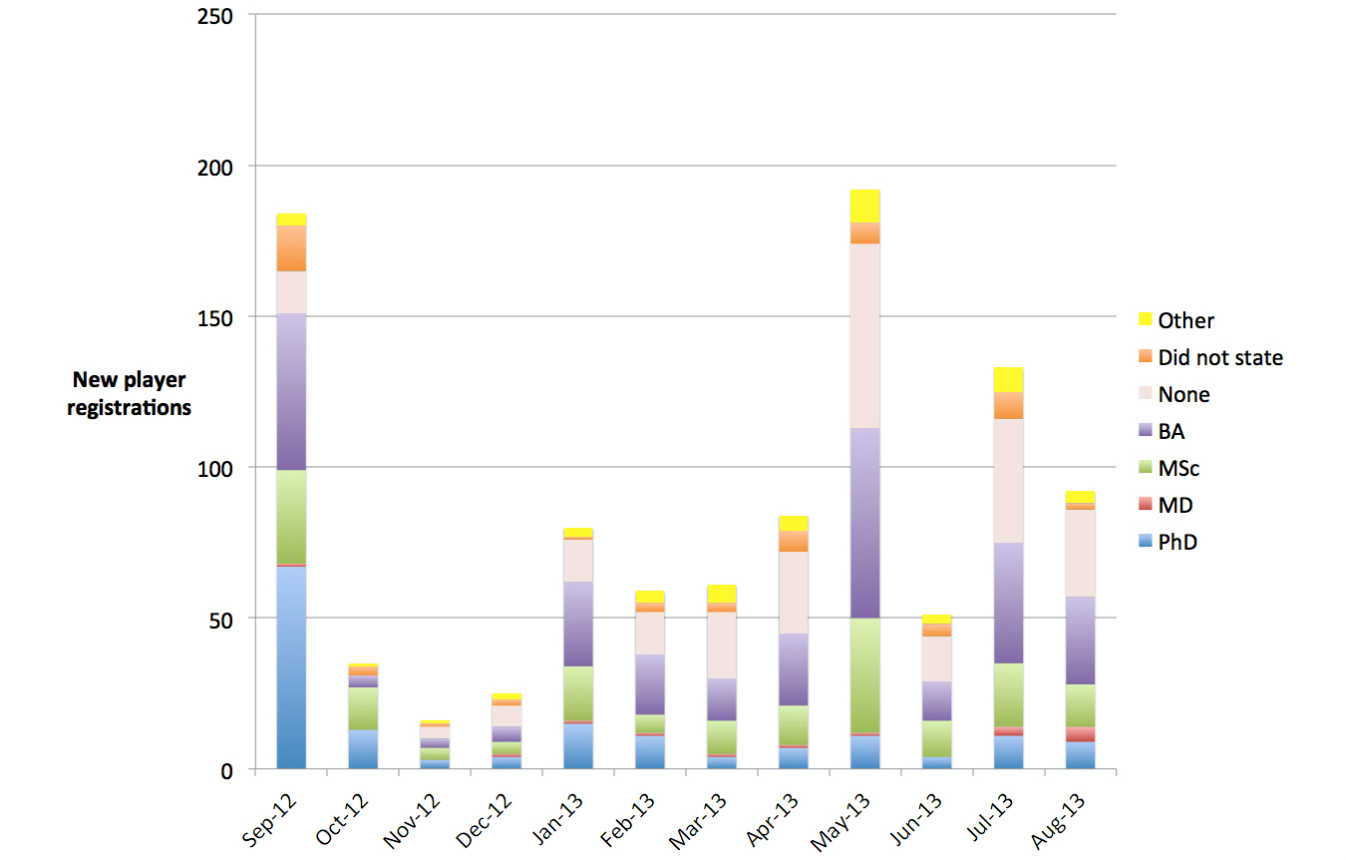
New player registrations per month, with academic degree. The figure shows the fluctuations in both the size and the demographics of the player population over time.

Over the course of the year, the number and demographics of players registering per month fluctuated (Figure 4 shows this fluctuation). In the first two months, 36.4% (67/184) and 37% (13/35) of the players who registered had PhDs. After those months, the percentage for the next four months ranged from 16% (4/25) to 18% (15/80), and then fluctuated between approximately 5% and 10% thereafter. We observed two notable spikes in player registrations. The first coincided with the launch of the game, its presentation at Genome Informatics 2012, and its advertisement as part of the Sage Bionetworks DREAM7 breast cancer prognosis challenge. The second, in May of 2013, is likely related to a posting on the popular website i09, which occurred on May 1, 2013 [21].

The total number of games played roughly followed the trends observed for new player registrations. The most games played on a single day were 550, on May 2, immediately after the i09 posting.

**Figure 5:**
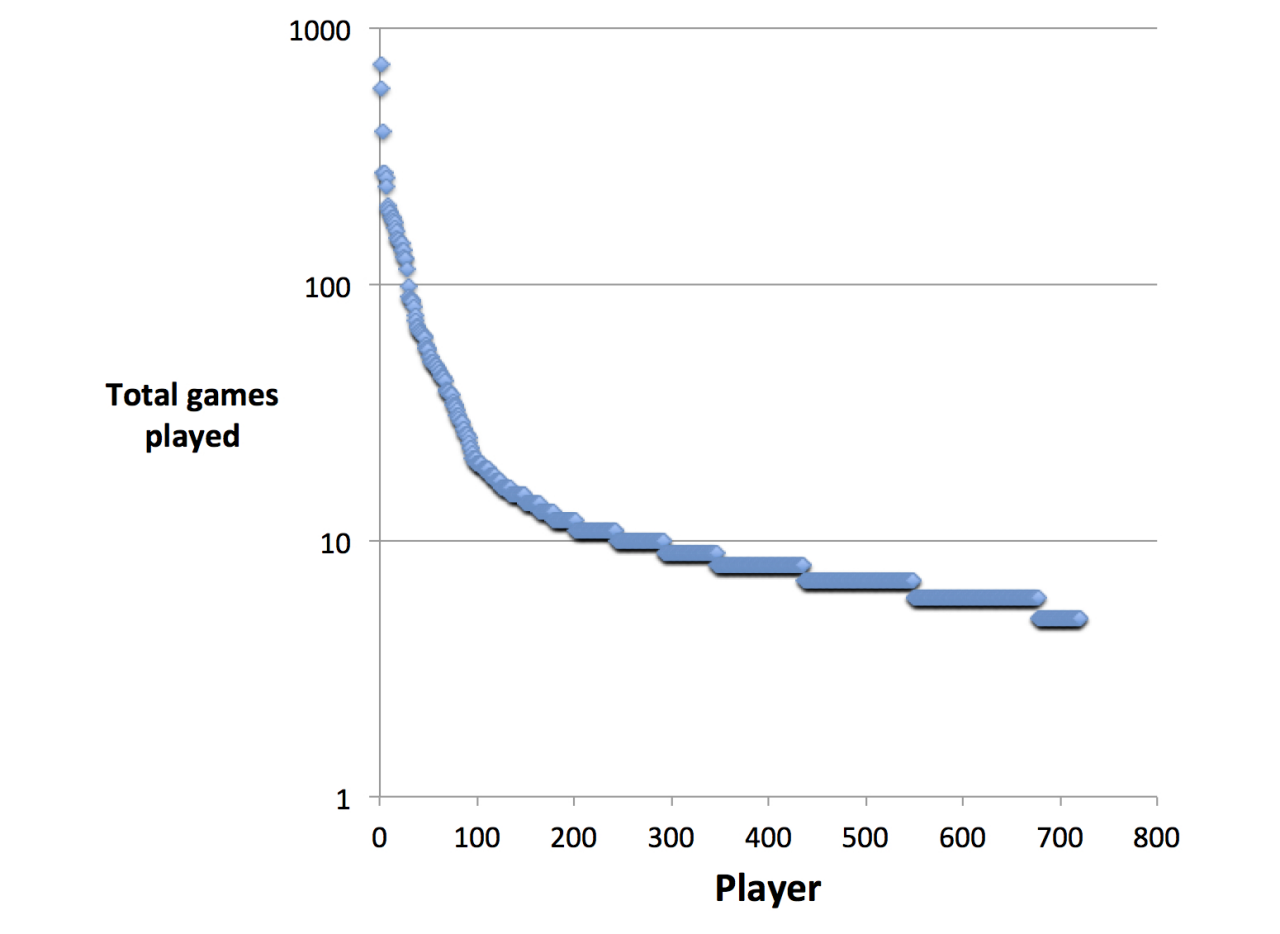
Games played per player. The majority of players only played a few games, while some players played several hundred games.

The number of games played per player followed a power law, consistent with most studies of the quantity of voluntary contributions in open environments (eg, Wikipedia contributions) [22]. There were 243 players that played more than 10 games, 28 players that played more than 100 games, and the most prolific player (“oneoff64”) that played 718 games (Figure 5 shows these numbers).

### Gene Set Evaluations

We evaluated three game collections: (1) “all”, (2) “expert”, and (3) “inexperienced”. “All” considers games from all players; “expert” includes games from players that indicated that they had either a PhD or an MD and knowledge of cancer; and “inexperienced” includes just the games played by people without an advanced degree, with no knowledge of cancer, and that were not biologists. Only the first five cards per player per board are used for the analysis. This reduces the chances that individual players who repeatedly play the same board, either through cheating or by repeatedly losing to Barney, can introduce bias based on overfitting that board. Each game should reflect only the player’s thoughts about the best genes for that board prior to seeing the results of the decision tree analysis.

For all the results reported here, we select genes with *P*≤.001 (see Aggregation Function in the Methods section). At that threshold, we observed 61 genes in the “all” group, 85 in the “expert” group, and 13 in the “inexperienced” group (Table 1). The complete set of game-derived gene rankings is available in Multimedia Appendix 3.

**Figure 6:**
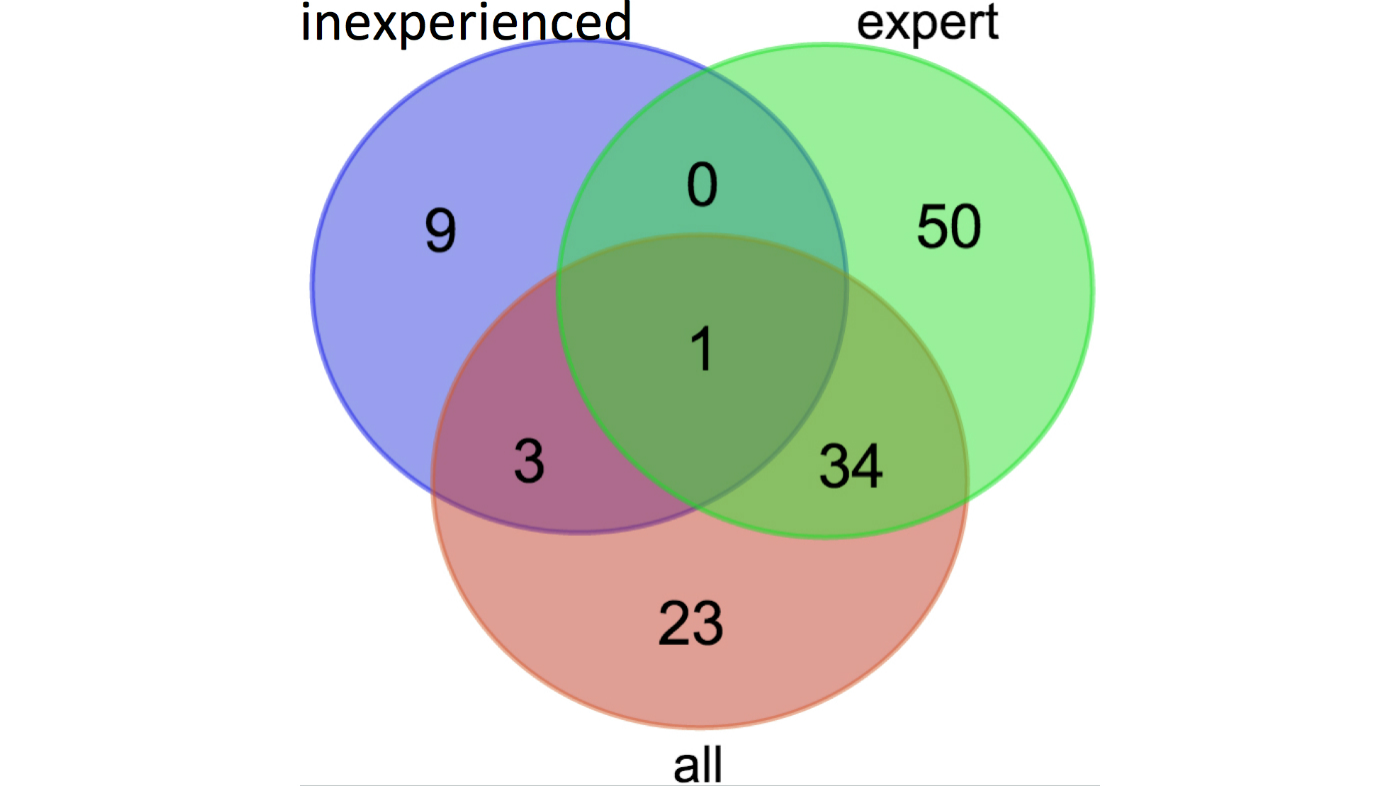
Overlap of game-derived gene sets.

There was one gene, CASP1, which appeared in all three sets. The “all” gene set included 35 genes that also appeared in the “expert” set, as well as 4 genes from the “inexperienced” set (Figure 6 shows these sets). Aside from CASP1, there was no overlap between the “expert” and “inexperienced” gene sets.

**Table 1:** Predictor gene sets derived from The Cure.

Player group	n genes *P*≤.001	Games considered	Contributing players
All	61	4314	477
Expert	85	1106	52
Inexperienced	13	1643	231

### Enrichment Analysis of Gene Sets

Given the gene sets identified above, the next question is whether or not they are relevant to breast cancer. Has knowledge successfully been transferred from the player population into the game? To address this question, we first used the WebGestalt enrichment analysis tool to identify which disease related terms were statistically overrepresented in the annotations of the genes [23]. We found both the “expert” gene set and the “all” gene sets to be significantly enriched for cancer-related diseases, while the “inexperienced” set was not significantly enriched for any diseases (Table 2). The background genes used for the enrichment analysis statistics corresponded to the 3731 genes that appeared in at least one game. The disease term with the most significant corrected *P* value in both the “all” and “expert” gene sets was “cancer or viral infections”. All of the top ten disease terms for both gene sets correspond to various kinds of cancer or cancer processes, such as “recurrence” and “disease progression”. Though they do not appear in the top ten results, “Breast neoplasms” and “Carcinoma, Ductal, Breast” are significantly represented in both gene sets (*P* < e-05).

**Table 2:** The top ten disease terms for the “expert” gene set based on WebGestalt analysis. All reported disease terms had adjusted *P* values using the Benjamini & Hochberg correction for multiple testing <.001. All of these terms were also significantly enriched in the “all” gene set with *P*<.001 except “Intestinal neoplasms“ which had a corrected *P* value of .01 in that set.

Disease term	Expert players (85 genes)		All (61 genes)	
	Genes in set	Adjusted *P* value (BH^a^)	Genes in set	Adjusted *P* value (BH^a^)
Cancer or viral infections	37	5.5e-16	25	8.1e-10
Neoplasms	32	4.7e-13	22	1.6e-08
Urogenital neoplasms	23	2.7e-11	12	9.0e-05
Cell transformation, neoplastic	16	4.7e-11	13	3.0e-09
Stomach neoplasms	14	2.6e-08	8	2.0e-04
Disease progression	16	3.7e-08	13	2.5e-07
Neoplastic processes	20	5.1e-08	18	1.3e-08
Recurrence	14	5.1e-08	11	1.1e-06
Intestinal neoplasms	15	6.1e-08	6	.01
Necrosis	15	1.1e-07	13	1.8e-07

^a^BH = Benjamini & Hochberg correction for multiple testing

### Comparison to Established Predictor Gene Sets

**Figure 7:**
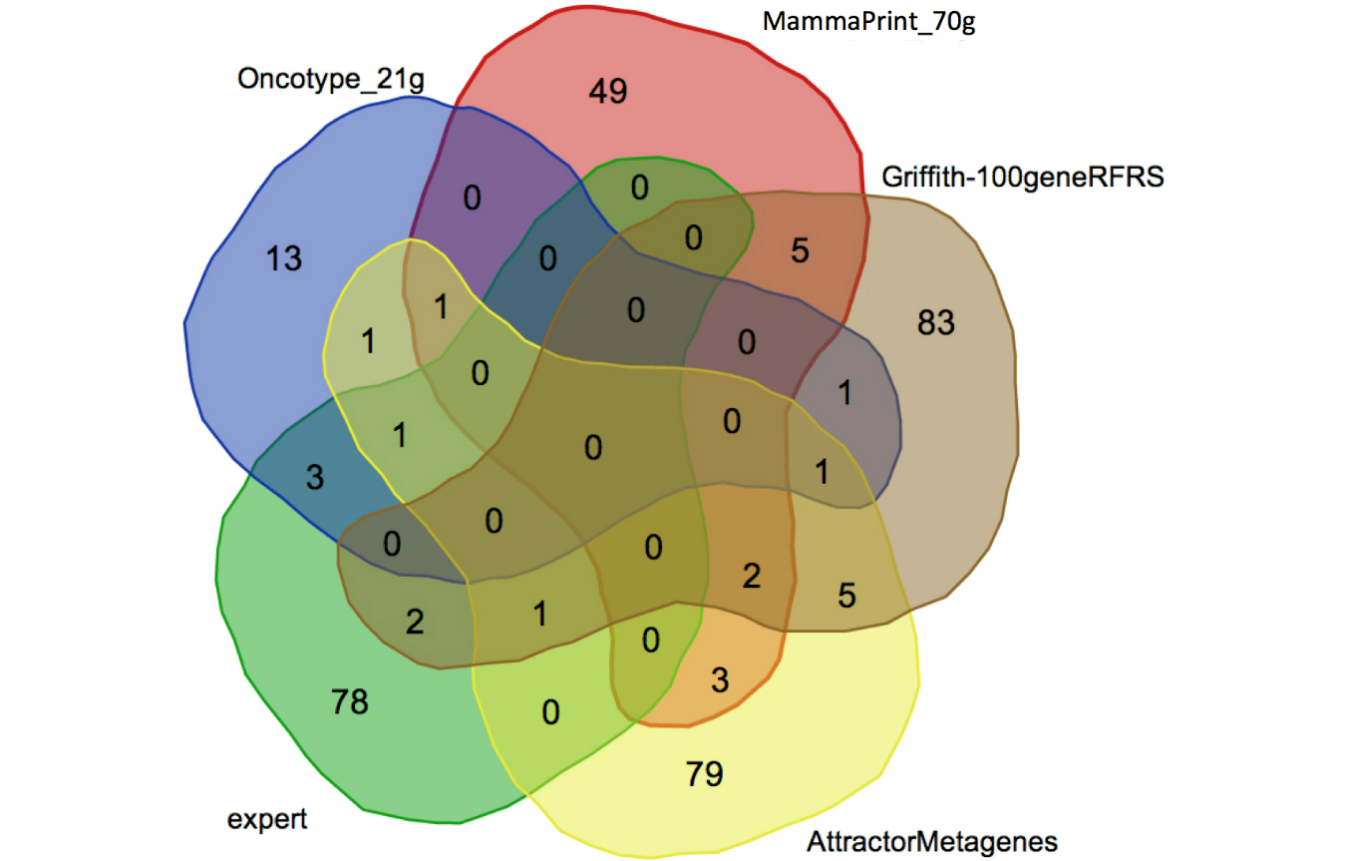
Overlap of "expert" gene set derived from game data (in green) with prior published predictor gene sets. RFRS: Random Forest Relapse Score.

In addition to the disease enrichment analysis, we measured the overlap between the game-derived gene sets and “gold standard” predictor gene sets used in commercial prognostic tests, and from recent publications. Figure 7 shows the overlaps between the expert game gene set, the 21 genes used in the OncoTypeDx test [24], the 70 genes in the MammaPrint test [2], the 100 genes recently identified via Random Forest analysis (RFRS) [4], and the 94 genes recently identified via the Attractor Metagenes approach [8]. Genes in the “gold standard” sets that never appeared in a played game were removed from the comparison (eg, only 58 of the 70 genes in the MammaPrint set were used.) The “expert” gene set contained four of the OncoType genes, zero of the MammaPrint genes, three of the RFRS genes, and two of the Attractor Metagenes. Based on a Fisher’s exact test, there was a statistically significant overlap with only the OncoType genes (*P*=2.026e-4).

### Classifier Evaluations

The gene set comparisons and enrichment analyses described above show clearly that the gene sets generated from the game data are nonrandom, with a significant representation of genes that are related to cancer. The final question addressed here is how well the game-derived gene sets do when used to create classifiers for predicting breast cancer survival.

**Figure 8:**
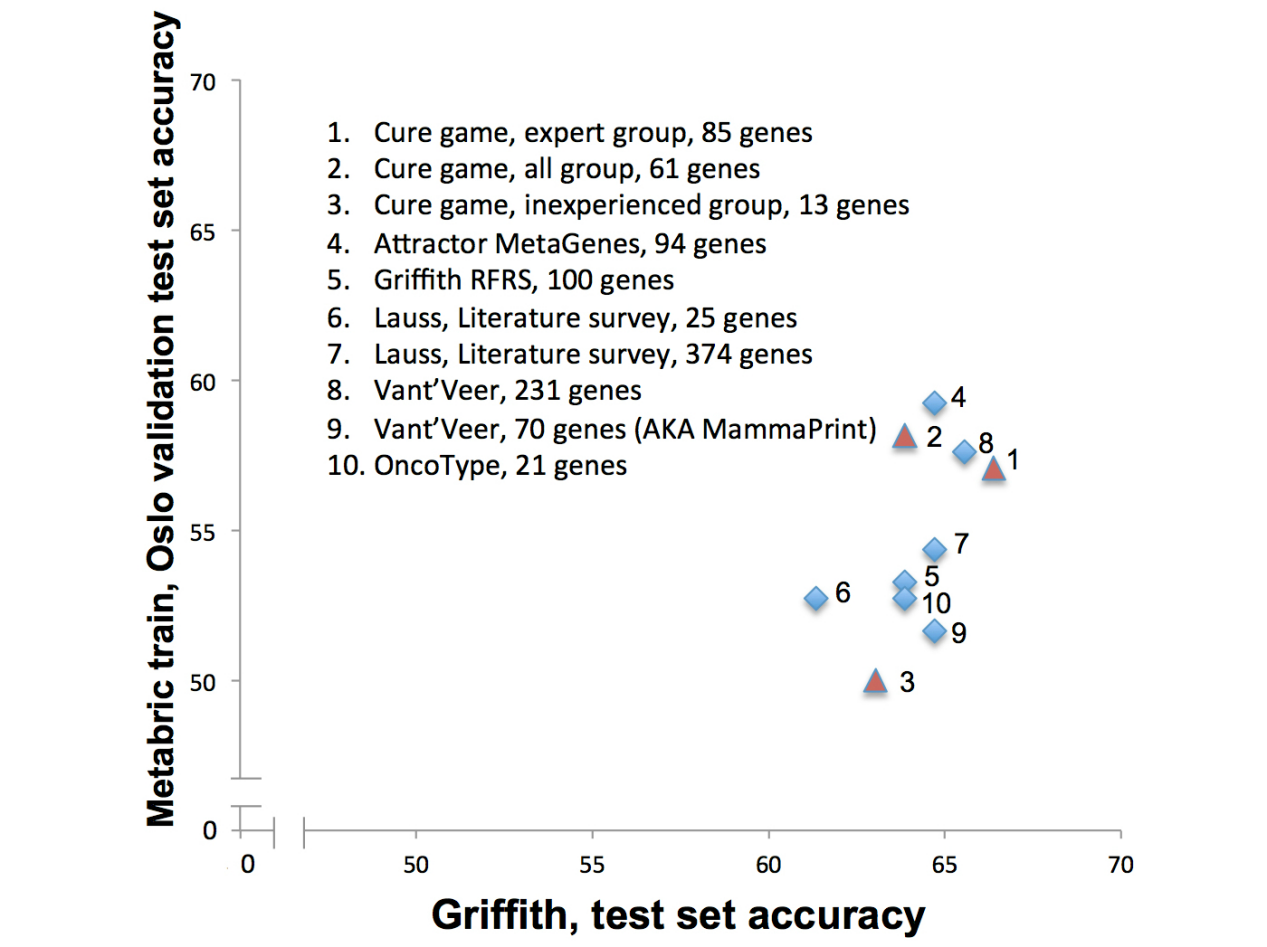
Evaluation of accuracy of models trained to predict ten year survival using gene sets derived from the game, and prior gene sets from the breast cancer literature. Lauss, Literature survey [27]. Vant’Veer datasets [3]. RFRS: Random Forest Relapse Score.

We conducted two experiments, each involving the development of machine learning models for predicting 10 year survival based only on gene expression information. In the first, we trained an SVM classifier using gene expression data from the Metabric dataset [25], and tested it on the Oslo validation dataset generated for the Sage Dream7 breast cancer challenge [3]. In the second, we used the dataset from [4], using the same division of training/test data described in that publication. In both cases, we varied only the gene sets provided to the classifiers, and measured the performance of each gene set based on the accuracy of the SVM on the samples in the corresponding test set. Figure 8 shows that both the “expert” and “all” gene sets from the game performed comparably to the OncoType, MammaPrint, RFRS, Attractor MetaGenes, and to gene sets selected in a literature review [26]. In fact, the “expert” gene set from The Cure had the highest accuracy on the Griffith test set, and the third highest accuracy on the Oslo test set. In contrast, the 13 genes selected by the “inexperienced” players produced the worst classifier for the Oslo test set, and the second worst for the Griffith test set.

Based on these experiments, and others employing different machine learning methods (data not reported), we could not establish a statistically significant difference between the performance of models trained using the game-derived gene sets versus models trained with gene sets from more established methodologies. While we could not prove that the game-derived “expert” gene set was better than the other gene sets in a statistically significant manner, none of the other gene sets—including those used in commercial tests—were found to be consistently better either. The lack of a clear “winner” in this analysis reinforces the concept that there are actually many different gene sets whose expression signatures are nearly equally predictive of a breast cancer prognosis [27]. Identifying the optimal combination of genes, clinical features (eg, age, lymph node status), and machine learning approach remains a future challenge.

### Player Survey

The Cure managed to attract and engage a surprisingly large number of people. To ascertain more about the player population, we conducted a survey of registered players as of November 2013. We sent an email to the 1162 players who had entered an email address when they registered, inviting them to answer questions about themselves, their motivations for playing, and their experience with the game. We received responses from 119 participants. While the respondents represent only about 10.24% (119/1162) of the total player population (and likely a more motivated segment), the responses do provide some interesting insights.

The first and perhaps most telling question in the survey was, “Why did you sign up for The Cure?”. Overall, 71.4% (85/119) indicated that they played to help breast cancer research, 52.9% (63/119) played to learn something, and just 43.7 (52/119) played in order to have fun. Respondents could select multiple answers. Given the design of The Cure website (“Play Games, Cure Cancer!”) [28], as well as the way it was promoted, it is surprising to see that the game aspect was actually the least motivational of the three. While we feel that developing this system as a game had a strong positive effect on recruitment and engagement, it is clear that there is a large pool of people that are highly motivated to contribute to breast cancer research in any way they can. The game was simply one more vehicle through which they could try to help. In some cases, this motivation is likely very personal, 63.6% (75/118) of respondents indicated that they know or have known someone that has or has had breast cancer.

**Figure 9:**
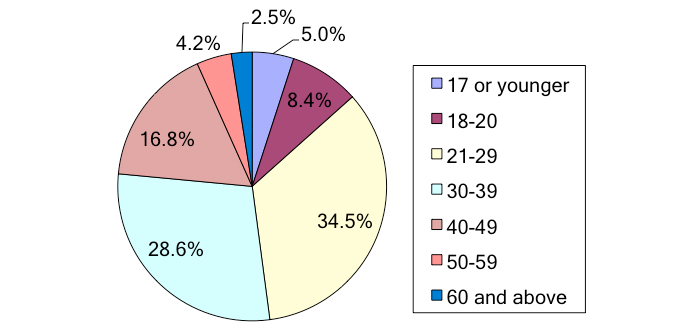
Ages of players.

Looking at the player demographics, we found that 59.0% (69/117) of the respondents were male, and 41.0% (48/117) female with 2 not responding. The largest age brackets were 21-29 years old (34.5%, 41/119) and 30-39 years old (28.6%, 34/119) (Figure 9 shows the ages).

**Figure 10:**
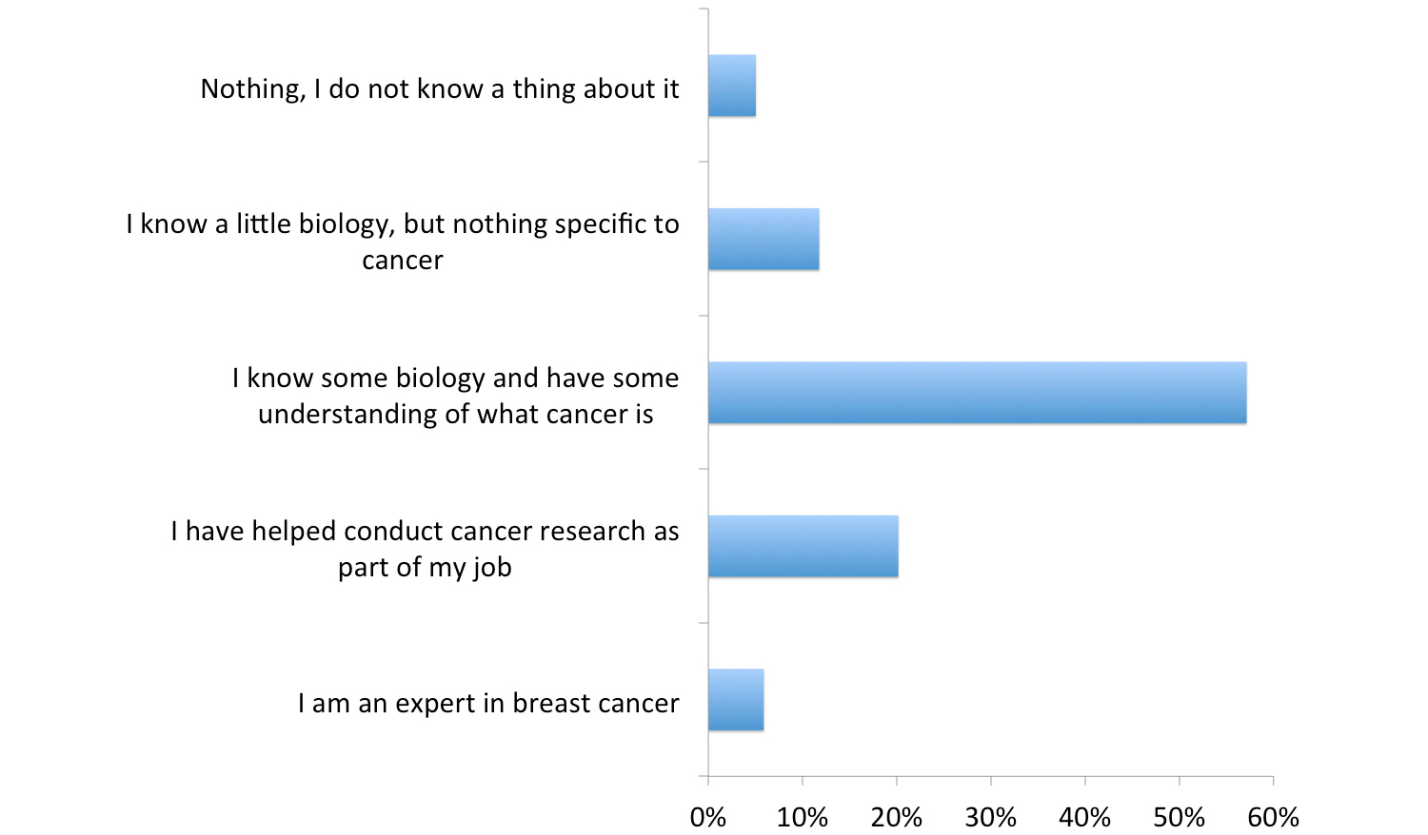
Levels of breast cancer knowledge among players.

Expanding on the expertise information collected when players registered, we asked the players to categorize their knowledge of breast cancer. The most popular answer by a wide margin was the middle expertise level, “I know some biology and have some understanding of what cancer is” at 57.1% (68/119) with numbers decreasing toward the high and low expertise levels (Figure 10 shows these answers).

Finally, we asked players whether the game was fun and whether or not they learned anything. Most (66.4%, 79/119) found the game to be “A little bit entertaining”, 14.3% (17/119) found it to be “very fun”, and 19.3% (23/119) found it “not at all” fun. The results for learning are similar, with 62.2% (74/119) feeling that they “learned a little bit”, 9.2% (11/119) that they “learned a lot”, and 28.6% (34/119) that they “did not learn thing”.

In summary, the survey showed that The Cure reached a demographically diverse audience containing both experts and novices, that most players found the game mildly entertaining and educational, and that the dominant motivation for playing was to help breast cancer research.

## Discussion

### Principal Results

The principal contribution of this work is as an exemplar to show that crowdsourcing games can be developed as a means to address problems that require expert-level knowledge. To our knowledge, this is the first serious game of this kind. In the introduction, we laid out three foundational question areas pertaining to: (1) recruiting participants, (2) capturing knowledge, and (3) translating the captured knowledge to advance a research objective. We now briefly explore each of these in turn.

While previous work on scientific discovery games such as Foldit have focused on visual problems that do not require any knowledge on the part of the participants, the task presented in The Cure was knowledge intensive. In order to successfully participate, players either had to bring significant prior experience or be willing to invest a substantial amount of time learning. Given the challenging nature of the task, and the importance of prior knowledge, the results from the first year of the game were quite positive in terms of recruitment (1000+ players) and engagement (nearly 10,000 games). Based on the data collected when players registered, and through the survey, the game clearly succeeded in capturing the attention of a diverse audience including both novices and experts. While motivations were mixed, the dominant theme appeared to be a strong desire to help advance breast cancer research.

The second question was whether or not knowledge could be captured from players. The gene rankings produced from both the entire set of games played (the “all” set) and the games played by the “expert” players clearly demonstrate that advanced biomedical knowledge was transferred from the community through the game (Table 2). The game succeeded in capturing knowledge from players with prior experience, but it did not appear to successfully harness the reading and reasoning ability of the nonexperts. This was apparent in the poor performance of the genes extracted from the “inexperienced” players (Figure 8).

The final question was whether or not the collected knowledge could be used to advance the state of the art in breast cancer survival prediction. While we demonstrated that the gene sets selected by the aggregated actions of the player community were both relevant and competitive with gene sets produced using other means, we did not conclusively generate a better predictor of breast cancer prognosis (Figure 8). There is no doubt that the case for applying serious games in the context of knowledge-intensive challenges would have been strengthened by a better result here. However, it is important to keep in mind that this is an extremely difficult, well studied problem that may not even have an optimal solution [27]. The fact that the gene sets derived from the game are comparable in terms of predictive accuracy to gene sets identified using statistical approaches backed by literally decades of research provides strong evidence that this approach is worthy of additional study.

### Limitations

The game described here was an early stage prototype with many limitations in terms of both its ability to achieve its purpose, and its ability to entertain players. Chief among the former was that the prebuilt boards severely constrained the number of different feature combinations that players could explore. The vast majority of possible gene sets simply could never be examined within this game framework. Further, because the aggregation function ranked individual genes rather than gene sets, it is unlikely that it would identify optimal feature combinations. In future iterations, it would be beneficial to adapt the game to allow advanced players more freedom to explore the feature space, while still maintaining the competitive dynamics that made the game entertaining.

Finally, the game could be made much more fun overall. The current formulation was highly repetitive, and had an extremely steep learning curve. The transition from the brief training stage to the real games was abrupt, and left many players confused. In the future, both the fun factor and the learning aspects of the game could be improved by implementing different levels of difficulty, providing more educational information in the early stages, and diversifying the tasks presented to players. Such changes should improve both player engagement and the quality of the information captured by the system. The code for The Cure game is open source, and we would warmly welcome any contributions or adaptations [29].

### Conclusions

There is a large, heterogeneous population of people on the Internet that actively seek ways to use their minds to help solve important problems. Games such as The Cure provide one avenue to tap into this hidden resource for biomedical discovery.

## Multimedia Appendix 1

Additional detail on methods. A text document containing additional information about the methods used to process the microarray datasets, select genes for boards, and execute the machine learning experiments.

## Multimedia Appendix 2

Cure database export. The complete SQL database used to capture data for The Cure game. The database contains: (1) gene annotation information provided to the players in the game, (2) gene sets used to compose the boards in the game, (3) genes (“cards”) selected by the players during their games including the order in which they were selected, (4) a log of games played, (5) logs of user actions including mouse click and hover events, and (6) player information (note that email and IP addresses have been removed). This is a compressed file that can be imported and opened using a MySQL database.

## Multimedia Appendix 3

Gene rankings from The Cure. An Excel workbook that shows the gene rankings generated from game play data. Rankings are shown for games from all players, from "experts" (those with a PhD or MD and knowledge of cancer), those with no expertise (no advanced degree, no knowledge of cancer, not a biologist), those with cancer knowledge, and those with a PhD or MD. Additional worksheets show the results from the WebGestalt analysis of the top ranked genes.

## Abbreviations

GWAP: games with a purpose
RFRS: Random Forest Relapse Score
SVM: support vector machine

## References

[1] Bray F, Ren JS, Masuyer E, Ferlay J (2013). Global estimates of cancer prevalence for 27 sites in the adult population in 2008. Int J Cancer.

[2] van 't Veer LJ, Dai H, van de Vijver MJ, He YD, Hart AA, Mao M, Peterse HL, van der Kooy K, Marton MJ, Witteveen AT, Schreiber GJ, Kerkhoven RM, Roberts C, Linsley PS, Bernards R, Friend SH (2002). Gene expression profiling predicts clinical outcome of breast cancer. Nature.

[3] Margolin AA, Bilal E, Huang E, Norman TC, Ottestad L, Mecham BH, Sauerwine B, Kellen MR, Mangravite LM, Furia MD, Vollan HK, Rueda OM, Guinney J, Deflaux NA, Hoff B, Schildwachter X, Russnes HG, Park D, Vang VO, Pirtle T, Youseff L, Citro C, Curtis C, Kristensen VN, Hellerstein J, Friend SH, Stolovitzky G, Aparicio S, Caldas C, Børresen-Dale AL (2013). Systematic analysis of challenge-driven improvements in molecular prognostic models for breast cancer. Sci Transl Med.

[4] Griffith OL, Pepin F, Enache OM, Heiser LM, Collisson EA, Spellman PT, Gray JW (2013). A robust prognostic signature for hormone-positive node-negative breast cancer. Genome Med.

[5] Daemen A, Griffith OL, Heiser LM, Wang NJ, Enache OM, Sanborn Z, Pepin F, Durinck S, Korkola JE, Griffith M, Hur JS, Huh N, Chung J, Cope L, Fackler MJ, Umbricht C, Sukumar S, Seth P, Sukhatme VP, Jakkula LR, Lu Y, Mills GB, Cho RJ, Collisson EA, van't Veer LJ, Spellman PT, Gray JW (2013). Modeling precision treatment of breast cancer. Genome Biol.

[6] Ross JS, Hatzis C, Symmans WF, Pusztai L, Hortobágyi GN (2008). Commercialized multigene predictors of clinical outcome for breast cancer. Oncologist.

[7] Weigelt B, Pusztai L, Ashworth A, Reis-Filho JS (2012). Challenges translating breast cancer gene signatures into the clinic. Nat Rev Clin Oncol.

[8] Cheng WY, Ou Yang TH, Anastassiou D (2013). Development of a prognostic model for breast cancer survival in an open challenge environment. Sci Transl Med.

[9] Dutkowski J, Ideker T (2011). Protein networks as logic functions in development and cancer. PLoS Comput Biol.

[10] Winter C, Kristiansen G, Kersting S, Roy J, Aust D, Knösel T, Rümmele P, Jahnke B, Hentrich V, Rückert F, Niedergethmann M, Weichert W, Bahra M, Schlitt HJ, Settmacher U, Friess H, Büchler M, Saeger HD, Schroeder M, Pilarsky C, Grützmann R (2012). Google goes cancer: Improving outcome prediction for cancer patients by network-based ranking of marker genes. PLoS Comput Biol.

[11] Bild AH, Yao G, Chang JT, Wang Q, Potti A, Chasse D, Joshi MB, Harpole D, Lancaster JM, Berchuck A, Olson JA, Marks JR, Dressman HK, West M, Nevins JR (2006). Oncogenic pathway signatures in human cancers as a guide to targeted therapies. Nature.

[12] Su J, Yoon BJ, Dougherty ER (2009). Accurate and reliable cancer classification based on probabilistic inference of pathway activity. PLoS One.

[13] Cheng WY, Ou Yang TH, Anastassiou D (2013). Biomolecular events in cancer revealed by attractor metagenes. PLoS Comput Biol.

[14] PubMed search for breast cancer.

[15] Good BM, Su AI (2013). Crowdsourcing for bioinformatics. Bioinformatics.

[16] von Ahn L, Dabbish L (2008). Designing games with a purpose. Commun. ACM.

[17] Cooper S, Khatib F, Treuille A, Barbero J, Lee J, Beenen M, Leaver-Fay A, Baker D, Popović Z, Players F (2010). Predicting protein structures with a multiplayer online game. Nature.

[18] Kawrykow A, Roumanis G, Kam A, Kwak D, Leung C, Wu C, Zarour E, Sarmenta L, Blanchette M, Waldispühl J, Phylo players (2012). Phylo: A citizen science approach for improving multiple sequence alignment. PLoS One.

[19] Luengo-Oroz MA, Arranz A, Frean J (2012). Crowdsourcing malaria parasite quantification: An online game for analyzing images of infected thick blood smears. J Med Internet Res.

[20] Kohavi R, John GH (1997). Wrappers for feature subset selection. Artificial Intelligence.

[21] Yesilbas A io9.

[22] Javanmardi S, Ganjisaffar Y, Lopes C, Baldi P (2009). User contribution and trust in wikipedia. Collaborative Computing: Networking, Applications and Worksharing.

[23] Wang J, Duncan D, Shi Z, Zhang B (2013). Web-based gene set analysis toolkit (WebGestalt): Update 2013. Nucleic Acids Res.

[24] Paik S (2007). Development and clinical utility of a 21-gene recurrence score prognostic assay in patients with early breast cancer treated with tamoxifen. Oncologist.

[25] Curtis C, Shah SP, Chin SF, Turashvili G, Rueda OM, Dunning MJ, Speed D, Lynch AG, Samarajiwa S, Yuan Y, Gräf S, Ha G, Haffari G, Bashashati A, Russell R, McKinney S, Langerød A, Green A, Provenzano E, Wishart G, Pinder S, Watson P, Markowetz F, Murphy L, Ellis I, Purushotham A, Børresen-Dale AL, Brenton JD, Tavaré S, Caldas C, Aparicio S, METABRIC Group (2012). The genomic and transcriptomic architecture of 2,000 breast tumours reveals novel subgroups. Nature.

[26] Lauss M, Kriegner A, Vierlinger K, Visne I, Yildiz A, Dilaveroglu E, Noehammer C (2008). Consensus genes of the literature to predict breast cancer recurrence. Breast Cancer Res Treat.

[27] Venet D, Dumont JE, Detours V (2011). Most random gene expression signatures are significantly associated with breast cancer outcome. PLoS Comput Biol.

[28] The Cure game website.

[29] Open source code repository for The Cure.

