# Supplementary material for: The Cure: Design and Evaluation of a Crowdsourcing Game for Gene Selection for Breast Cancer Survival Prediction
Source: JMIR Serious Games. 2014 Jul 29;2(2):e7. doi: 10.2196/games.3350 (PMC4307816; doi:10.2196/games.3350)
Supplement: Supplementary file 1 [file games_v2i2e7_app1.pdf]

Additional details on methods for "The Cure: Making a game of gene selection for breast cancer survival prediction"

Composing gene sets for four rounds of game play

The game play data presented here was collected in four distinct rounds, with each round consisting of a set of 100 boards (sets of 25 distinct genes). We chose gene sets for boards by first identifying a list of 2500 'interesting' genes based on unsupervised analysis of a genomic dataset. We then sampled randomly from this gene list to produce the boards for the game.

Dataset used for rounds 1 and 2 (Sage DREAM7 challenge)

The first two rounds corresponded to the two iterations of the training data provided for the Sage DREAM7 challenge [1]. In both cases Sage provided a processed subset of the METABRIC dataset [2] with information about gene expression, copy number variation (CNV), and clinical features including survival information. We used the survival data to group the samples into two classes: those with less than ten-year survival from the point of diagnosis and those with greater than ten-year survival. For these data sets we selected genes to include in the game based on both gene expression information and CNV data. First we used the CNV information to rank the genes based on the sum of squares across all samples and selected the top 1000 genes. Next, we developed a ranking of the genes based on expression data as follows:

1. Remove probes with poor matches to genes based on the ReMOAT annotation data [3].
2. Remove probe sets where the maximum expression value across all samples is lower than the overall median
3. Rank probe sets based on their variance across all samples

Finally, we merged the genes selected based on CNV information with the expression-based ranking to produce a single table with 2500 unique genes.

Dataset used for rounds 3 and 4 (Griffith dataset)

Rounds three and four used the meta gene expression dataset assembled for [4]. For this dataset, genes were selected following the same approach as described in the original publication (where at least 20% of samples should have intensities greater than the background threshold and the coefficient of variation is between 0.7 and 10). Genes that passed those filters were then ranked based on their variance across all samples to produce a set of 2500 unique genes.

Composing boards

In the first round, we seeded each board with one gene with a high ReliefF [5] value based on the combined CNV and expression data with all other genes selected randomly from the set of 2500. (Note that players did not preferentially select the seeded genes.) In the three subsequent rounds, we first created 50 boards by randomly sampling from the 2500. Then we selected the second set of 50 boards by

sampling from the genes used in the first 50 boards such that each gene appears in two boards per round. While reducing the coverage of genes, this strategy allowed players to assess each gene in multiple contexts, hopefully allowing a fairer assessment of the gene's overall value. Each round had some overlap in terms of genes used. In total, 3,731 distinct genes were used in boards played in the game.

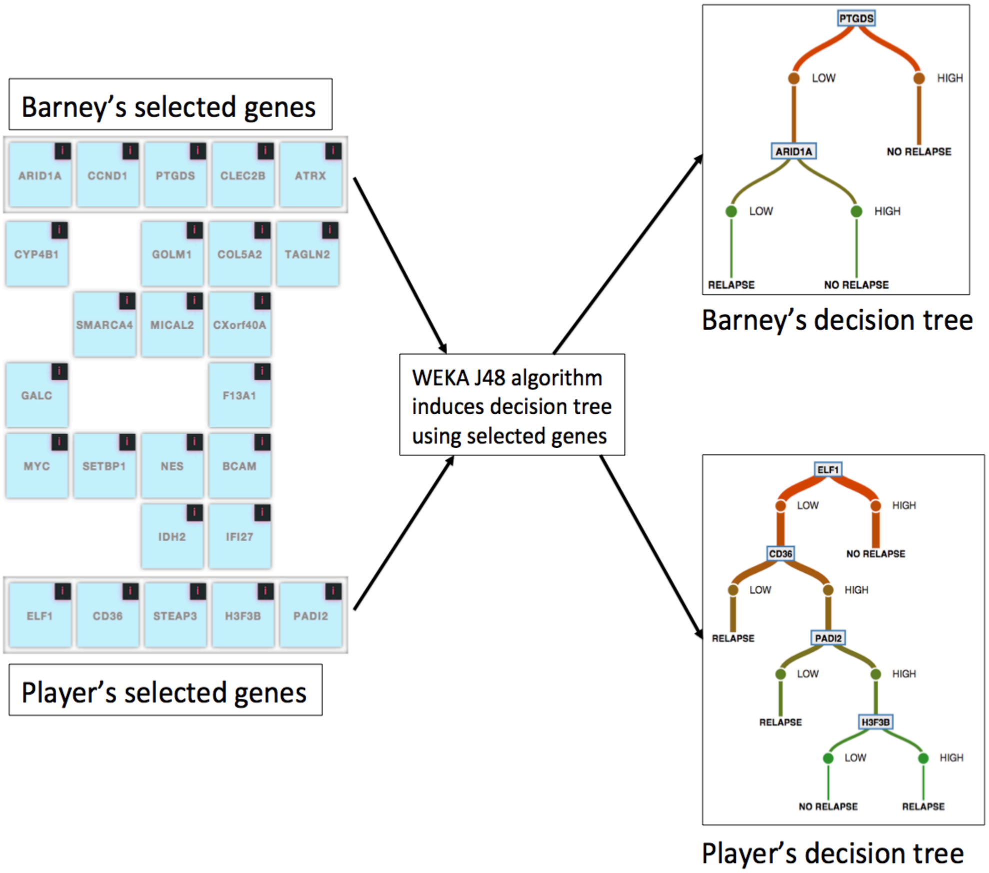

Fig. 1. Use of WEKA to construct decision trees using the genes selected by the players. As each gene is added to the player's or the automated opponent Barney's hand, the genes in the hand are sent to the server. On the server, WEKA is used to construct and evaluate decision trees that predict ten-year survival based on genomic measurements of the selected gene's activities in samples from patients that did and did not survive beyond ten years. For example, Barney's decision tree depicted here reads as "if PTGDS expression is high, then predict NO RELAPSE (>10 year survival), else if ARID1A expression is low then predict RELAPSE (<10 year survival) else if ARID1A expression is high then predict NO REPLASE (>10 year survival). The tree shown to the player is constructed using all available training instances. Note that not all genes in a hand may be selected by the algorithm to appear in the tree. The score used in the game was the average accuracy of decision trees constructed using the selected genes in a 10-fold cross-validation experiment. Barney's genes were chosen randomly.

Scoring hands

Each time a gene was added to a player's hand during a game, the server immediately responded with a score for the genes in their hand and a decision tree inferred using those genes (Figure 1). This was accomplished using source code adapted from the Waikato Environment for Knowledge Analysis (WEKA) [6]. We used WEKA's implementation of the C4.5 decision tree induction algorithm (called J48) as well as its code for cross-validation. In cross-validation, learning models are

trained on random subsets of the data and tested on the held out samples. The scores displayed in the game were accuracy estimates from 10-fold cross-validation experiments. The trees shown were inferred using the provided genes and all training data. Trees were constructed based on both CNV and expression data for round 1, and just expression data for rounds 2-4.

Classifier evaluations

We used WEKA's default implementation of the sequential minimal optimization algorithm for training the SVMs used in the gene set analysis.

References

1. Margolin, A.A., et al., Systematic analysis of challenge-driven improvements in molecular prognostic models for breast cancer. Sci Transl Med, 2013. 5(181): p. 181re1.
2. Curtis, C., et al., The genomic and transcriptomic architecture of 2,000 breast tumours reveals novel subgroups. Nature, 2012. 486(7403): p. 346-52.
3. Barbosa-Morais, N.L., et al., A re-annotation pipeline for Illumina BeadArrays: improving the interpretation of gene expression data. Nucleic Acids Res, 2010. 38(3): p. e17.
4. Griffith, O.L., et al., A robust prognostic signature for hormone-positive node-negative breast cancer. Genome Med, 2013. 5(10): p. 92.
5. Kononenko, I. Estimating attributes: analysis and extensions of RELIEF. in Machine Learning: ECML-94. 1994. Springer.
6. Witten, I.H., et al., Weka: Practical machine learning tools and techniques with Java implementations. 1999.
